# A Gemini Cationic Lipid with Histidine Residues as a Novel Lipid-Based Gene Nanocarrier: A Biophysical and Biochemical Study

**DOI:** 10.3390/nano8121061

**Published:** 2018-12-16

**Authors:** María Martínez-Negro, Laura Blanco-Fernández, Paolo M. Tentori, Lourdes Pérez, Aurora Pinazo, Conchita Tros de Ilarduya, Emilio Aicart, Elena Junquera

**Affiliations:** 1 Grupo de Química Coloidal y Supramolecular, Departamento de Química Física, Facultad de Ciencias Químicas, Universidad Complutense de Madrid, 28040 Madrid, Spain; mmnegro@ucm.es (M.M.-N.); paolo.tentori@sns.it (P.M.T.); aicart@ucm.es (E.A.); 2 Departamento de Farmacia y Tecnología Farmacéutica, Facultad de Farmacia, Universidad de Navarra, Instituto de Investigación Sanitaria de Navarra (IdiSNA), 31008 Pamplona, Spain; lblancof@unav.es (L.B.-F.); ctros@unav.es (C.T.d.I.); 3 Dpto. Tecnología Química y Tensioactivos, IQAC-CSIC, 08034 Barcelona, Spain; lpmste@cid.csic.es (L.P.); apgste@cid.csic.es (A.P.)

**Keywords:** lipid-based gene nanocarrier, gemini cationic lipid with histidine residues, gene delivery, plasmid DNAs, transfection, cell viability, biophysical characterization

## Abstract

This work reports the synthesis of a novel gemini cationic lipid that incorporates two histidine-type head groups (C_3_(C_16_His)_2_). Mixed with a helper lipid 1,2-dioleoyl-*sn*-glycero-3-phosphatidyl ethanol amine (DOPE), it was used to transfect three different types of plasmid DNA: one encoding the green fluorescence protein (pEGFP-C3), one encoding a luciferase (pCMV-Luc), and a therapeutic anti-tumoral agent encoding interleukin-12 (pCMV-IL12). Complementary biophysical experiments (zeta potential, gel electrophoresis, small-angle X-ray scattering (SAXS), and fluorescence anisotropy) and biological studies (FACS, luminometry, and cytotoxicity) of these C_3_(C_16_His)_2_/DOPE-pDNA lipoplexes provided vast insight into their outcomes as gene carriers. They were found to efficiently compact and protect pDNA against DNase I degradation by forming nanoaggregates of 120–290 nm in size, which were further characterized as very fluidic lamellar structures based in a sandwich-type phase, with alternating layers of mixed lipids and an aqueous monolayer where the pDNA and counterions are located. The optimum formulations of these nanoaggregates were able to transfect the pDNAs into COS-7 and HeLa cells with high cell viability, comparable or superior to that of the standard Lipo2000*. The vast amount of information collected from the in vitro studies points to this histidine-based lipid nanocarrier as a potentially interesting candidate for future in vivo studies investigating specific gene therapies.

## 1. Introduction

Amino acids play important roles in cell life, acting as cell signaling molecules, regulating gene expression, and synthesizing hormones, among others. These features make them attractive as components of amino acid-based gene carriers for gene therapy, or as a part of different kind of molecules, such as of cationic monovalent or multivalent lipids [1,2,3,4,5,6], polypeptides [7,8,9,10,11], polymers [12,13,14,15], polycations [16] and/or dendrimers [11,17,18,19,20]. Generally, gene therapy has focused on viral and non-viral vectors for the treatment of hereditary or acquired illnesses [21,22,23]. In the case of viral carriers, the positively charged amino acids in the virus capsids are the basis for the tremendous success of this class of vectors in cell penetration [24]. However, they can easily trigger both innate and adaptive immune responses, which justifies the vast efforts made towards the synthesis of non-viral vectors [25,26].

Among other non-viral carriers, cationic lipids (CLs) constitute adequate platforms for effective gene compaction [27,28,29,30]. In addition, they show interesting features such as ease of manufacture, low cost, and biocompatibility; however, their low in vivo delivery efficiency has fueled the search for new cationic vectors. For instance, gemini cationic lipids (GCLs), structurally formed by two hydrophobic tails and two cationic head groups linked by a spacer, have attracted notable attention [27,31]. A variety of modifications to their structure can improve the transfection efficiency and reduce the cellular toxicity [30,32,33]. In this context, amino acid derivatives have been studied not only in cationic lipids forming lipoplexes [1,3,5,34,35,36,37,38], but also in polyplexes [5,12,39], to enhance the efficiency of gene delivery. For example, lysine-rich segments can facilitate the condensation of DNA [40]. The use of lysine units as cationic groups leads to interesting chiral nanostructures (i.e., ribbon-type and nanotubes) that can also thoroughly condense and compact the DNA, making them suitable for implementation as gene delivery vectors [40,41]. Arginine-based lipids also exhibit relevant DNA binding capability through parallel hydrogen bonding of the guanidinium group present in arginine [42,43,44]. Importantly, due to their ability to disrupt endosomes via protonation of the imidazole group at physiological pH, lipids comprising histidine residues yield high gene compaction rates, together with prominent transfection efficiencies [5,38,45,46,47,48]. Double-chain imidazolium surfactants [28,49], also exhibit effective siRNA delivery capacity, while gemini imidazolium surfactants efficiently condense DNA and deliver nucleotides inside human cells [33,50,51]. 

**Scheme 1:**
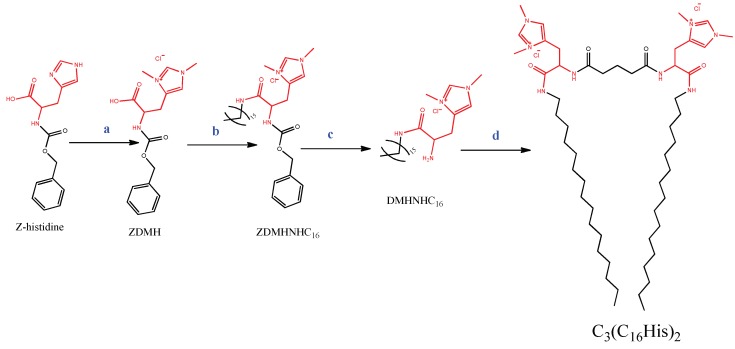
Chemical procedure used to synthesize the gemini cationic lipid C_3_(C_16_His)_2_ from *N*(α)-Cbz-histidine: (**a**) (CH_3_)_2_SO_4_ in methanol, (**b**) hexadecyl amine, benzotriazol-1-yloxy)tris(dimethylamino)phosphonium hexafluorophosphate (BOP), and 1,4-diazabicyclo[2.2.2]octane (DABCO) in dimethyl formamide (DMF), (**c**) hydrogenation, Pd/C in methanol, and (**d**) glutaric acid (HOOC-CH_2_-CH_2_-CH_2_-COOH), BOP, and DABCO in DCM.

However, the use of a cationic nanocarrier of plasmid DNA, combining the capability of gemini-type lipids to transfect nucleic acids efficiently together with the high biocompatibility of the amino acid histidine head groups, remains unexplored and thus, was the objective of the present work. The study demonstrates the potential for gene delivery of a cationic gemini lipid, comprised of two *N*(τ),*N*(π)-bis(methyl)-histidine hexadecyl amides linked through a spacer chain of three methylene groups (referred to as C_3_(C_16_His)_2_) (see Scheme 1). In order to evaluate these features, the C_3_(C_16_His)_2_ gemini cationic lipid was mixed with 1,2-dioleoyl-*sn*-glycero-3-phosphatidyl ethanol amine (DOPE), a well-known fusogenic helper lipid. Firstly, physicochemical characterization was carried out for C_3_(C_16_His)_2_/DOPE-pDNA lipoplexes containing two different DNA plasmids (pDNA), one encoding the green fluorescent protein (GFP; pEGFP-C3) and the other a luciferase (pCMV-Luc). Agarose gel electrophoresis and zeta potential analyses were used to evaluate the compaction of both plasmids, while their structure was characterized by small-angle X-ray scattering (SAXS) and the bilayer fluidity by fluorescence anisotropy. The transfection efficiency was evaluated in vitro using the COS-7 and HeLa cell lines, through fluorescence assisted cell sorting (FACS) and luminometry. More interestingly, the C_3_(C_16_His)_2_/DOPE-pDNA lipoplexes were found to exhibit little cytotoxicity across all the formulations studied in the present work. With the aim to corroborate the potential of this GCL bearing histidine residues as a therapeutic vector, further in vitro experiments were performed with a therapeutic plasmid (pCMV-IL12) encoding IL-12, a heterodimeric cytokine linked with autoimmunity. The biological in vitro results demonstrate that the C_3_(C_16_His)_2_/DOPE-pDNA lipoplexes may have an appreciable potential as efficient and biocompatible nucleic acid nanocarriers. Nonetheless, further in vivo experiments are necessary to confirm them as promising therapeutic vectors.

## 2. Materials and Methods

### 2.1. Materials 

The divalent gemini cationic lipid, bis(*N*(*τ*),*N*(*π*)-bis(methyl)-histidine hexadecyl amide) propane (C_3_(C_16_His)_2_), was synthesized according to a procedure fully detailed in the Supplementary Materials. The remaining compounds, all of the highest grade commercially available and used without further purification, were supplied by the following manufacturers: hexadecyl amine by Fluka (Bucharest, Romania); *N*(α)-carbobenzyloxy-l-histidine (*N*-Cbz-l-His.HCl) by Novabiochem AG (Laufelfingen, Switzerland); trifluoroacetic acid (TFA) and palladium on activated charcoal (Pd/C, 10%) by Merck (Darmstadt, Germany); 1,4-diazabicyclo[2.2.2]octane (DABCO) by Aldrich (Saint Louis, MO, USA); (benzotriazol-1-yloxy)tris(dimethylamino)phosphonium hexafluorophosphate (BOP) by TCI Europe (Zwijndrecht, Belgium); deuterated solvents by Eurisotop (Saint-Aubin, France); the zwitterionic lipid 1,2-dioleoyl-*sn*-glycero-3-phosphatidyl ethanol amine (DOPE) by Avanti Polar Lipids, Inc., (Alabaster, AL, USA); and the sodium salt of calf thymus DNA (ctDNA) by Sigma-Aldrich (St. Louis, MI, USA). The pEGFP-C3 plasmid DNA (4700 *bp*) encoding GFP was extracted from competent *Escherichia coli* bacteria previously transformed with pEGFP-C3 using a GenElute HP Select Plasmid Gigaprep Kit (Sigma-Aldrich). The pCMV-Luc VR1216 plasmid DNA (6934 *bp*) encoding a luciferase (Clontech, Palo Alto, CA, USA) was amplified in *E. coli* using a Qiagen Plasmid Giga Kit (Qiagen GMBH, Hilden, Germany). The plasmid pCMV-interleukin-12 (5500 *bp*) encoding interleukin-12 (pCMV-IL12) was kindly provided by Dr. Chen Qian, (University of Navarra, Pamplona, Spain). Solutions were prepared with distilled water from a Milli-Q Millipore system.

### 2.2. Characterization of the Intermediates and the Gemini Cationic Lipid C_3_(C_16_His)_2_

The structures of the target compounds were characterized by ^1^H and ^13^C NMR (nuclear magnetic resonance spectroscopy). Chemical shifts (*δ*) are reported in parts per million (ppm) downfield from tetramethylsilane (TMS), and specific details are given in the Supplementary Materials. Distortionless enhancement by polarization transfer (DEPT) spectra was recorded to phase up and down the CH/CH_3_ and CH_2_ signals, respectively. Mass spectra with fast atom bombardment (FAB) or electrospray techniques were recorded on a VG-QUATTRO. The determination of Fourier transformation infrared spectroscopy (FT-IR) was performed on a Nicolet FT-IR (Avatar 360 equipped with a Smart iTR accessory) with spectral range 4000–4500 cm^−1^. Characterization of the C_3_(C_16_His)_2_ gemini cationic lipid by ^1^H, ^13^C, and ^13^C-DEPT NMR spectra, and the FT-IR and UPLC-MS profiles, are also collected in the Supplementary Materials.

### 2.3. Preparation of Lipoplexes

Firstly, in order to prepare a liposomal gene nanocarrier in (4-(2-hydroxyethyl)-1-piperazineethanesulfonic acid) HEPES solution, following a procedure fully described earlier [32], appropriate amounts of C_3_(C_16_His)_2_ and DOPE were dissolved in chloroform to get the desired molar fractions of the cationic lipid (*α*) in the lipid mixture. Briefly, after vortexing the lipid solution, chloroform was removed and the dried films were hydrated with 40 mM HEPES (*pH* 7.4), which had been homogenized by vortexing and sonication to obtain a multilamellar liposome solution and, following a sequential extrusion method, converted to unilamellar liposomes of ~100 nm size. The lipoplexes were obtained by adding appropriate amounts of pDNA to each mixed lipid solution [32,33]. The final pDNA concentrations to fit the optimum conditions for each experimental technique used in this work were selected as follows: 1 mg/mL for zeta potential, 0.1 mg/mL for fluorescence anisotropy, 200 μg/capillary (~5 mg/mL) for SAXS, and 1 μg/well (2 μg/mL) for in vitro studies.

### 2.4. Experimental Methods

*Zeta potential and particle size*: A phase analysis light scattering technique (Zeta PALS, Brookhaven Instruments Corp., Holtsville, NY, USA) was used to obtain the zeta potential of the samples, prepared with buffer 40 mM HEPES (pH 7.4) at 25 °C [52]. The particle size was obtained by a dynamic light scattering (DLS) method using a particle analyzer (Zeta Nano Series; Malvern Instruments, Barcelona, Spain) [52]. Each zeta potential and particle size data point was taken as the average of 50 and 30 independent measurements, respectively. Measurements were carried out as a function of the total lipid/DNA mass ratio (*m*_*L*^+^_ + *m*_*L*^0^_)/*m_pDNA_* (where *m*_*L*^+^_, *m*_*L*^0^_, and *m_DNA_* are the C_3_(C_16_His)_2_, DOPE, and pDNA mass, respectively), and at various molar fractions (*α*) of the cationic lipid in the C_3_(C_16_His)_2_/DOPE mixed lipid.

*DNA compaction assay by gel electrophoresis*: Agarose gel electrophoresis experiments using a Gel Doc XR instrument (Bio-Rad) were carried out to analyze the compaction of pDNA by the mixed lipid nanocarrier. Details and experimental conditions of the procedure are reported previously [52]. Lipoplexes were loaded on 1% agarose gels (with 0.7 μL of GelRed). Samples were excited at 302–312 nm, and the fluorescence spectra emitted were collected at 600 nm. The fluorescence intensity was measured using the commercial Quantity One software.

*DNA protection assay by gel electrophoresis*: The same Gel Doc XR instrument (Bio-Rad) was used in gel electrophoresis experiments [52] to analyze the pDNA protection degree against degradation by DNase I when pDNA was forming the C_3_(C_16_His)_2_/DOPE-pDNA lipoplex. DNase I (1 U/µg of pDNA) was added to each lipoplex solution. Then, 20 µL of 0.25 M EDTA was added to inactive DNase I and the samples were incubated for 15 min. Next, 15 µL of 25% sodium dodecyl sulphate (SDS) was added to disrupt the lipoplexes, and later the samples were incubated for 5 min. The solutions were electrophoresed for 40 min under 80 mV, in 1% agarose gel with 1 μL of EtBr. Samples were excited at 482 nm, and the fluorescence spectra emitted were collected at 616 nm.

*Small-angle X-ray scattering*: SAXS experiments were carried out on the beamline NCD11 at the ALBA Synchrotron (Barcelona, Spain). Details of the experiment and preparation of samples were fully described recently [52]. The energy of the incident beam was 12.6 KeV (*λ* = 0.995 *Å*). Samples were placed in sealed glass capillaries with an outside diameter of 1.5 mm. The scattered X-rays were detected on a Quantum 210r CCD detector, converted to one-dimensional scattering by radial averaging, and represented as a function of the momentum transfer vector (*q* = 4πsin*θ*/*λ*, where *θ* is half the scattering angle and *λ* is the wavelength of the incident X-ray beam). SAXS experiments were performed for C_3_(C_16_His)_2_/DOPE-pDNA lipoplexes at an effective charge ratio (C_3_(C_16_His)_2_/DNA) of *ρ_eff_* = 4, and at various cationic lipid molar fractions of the mixed lipid (*α*).

*Fluorescence anisotropy*: Fluorescence anisotropy of the 1,6-diphenylhexatriene (DPH) probe was measured on a Perkin Elmer LS-50B luminescence spectrometer following an experimental protocol described elsewhere [53]. The probe, included in the mixed lipid bilayer, was excited at 360 nm, and its fluorescence emission was collected at 430 nm with slit widths of 2.5 nm. The anisotropy values, $r\;=\left(I_{VV}-GI_{VH}\right)/\left(I_{VV\;}+2GI_{VH}\right)$, were determined by measuring the intensity of the light emitted by the DPH probe, with the excitation and emission polarized following the modes: vertical–vertical ($I_{VV})$, vertical–horizontal ($I_{VH}$), horizontal–horizontal ($I_{HH})$, and horizontal–vertical ($I_{HV})$. The instrument grating factor $(G=I_{HV}/I_{HH}$) allowed for the correction of optical and electronic differences in the parallel and perpendicular channels was estimated as the average of 10 measurements for each solution.

*Cell culture*: COS-7 (african green monkey kidney) and HeLa (human cervix adenocarcinoma) cells, supplied by American Type Collection (Rockville, MD, USA), were used to carry out in vitro biological experiments (transfection and cell viability). Experimental conditions of the complete medium, including fetal bovine serum (FBS), were similar to those detailed in earlier studies [52].

*In vitro Transfection efficiency*: The transfection efficiency of the C_3_(C_16_His)_2_/DOPE-pDNA lipoplex formulations was analyzed through two complementary methods: luminometry for the pDNAs (pCMV-Luc and pCMV-IL12) encoding luciferase, and fluorescence assisted cell sorting (FACS) for the pDNA (pEGFP-C3) encoding GFP. In both methods [52], each measurement was carried out in triplicate in three wells, from three independent cultures (~50,000 cells/cm^2^). Lipofectamine (Lipo2000*) was used as the positive control.

*Luminometry*: A luminometer (Sirius-2, Berthold Detection Systems, Innogenetics, Diagnóstica y Terapéutica, Barcelona, Spain) was used to determine the luciferase activity of the plasmid pCMV-Luc, using the luciferase assay reagent Promega. The experimental conditions and the followed procedure, using 48-well-plates, were fully described earlier [52]. The protein content was determined with the DC protein assay reagent (Bio-Rad, Hercules, CA, USA). The luciferase activity of the plasmid pCMV-IL12 was determined with an ELISA kit for murine IL12p70 (BD OptEIA ELISA sets, Pharmingen, San Diego, CA, USA) according to the manufacturer’s instructions. In both studies, data were obtained in *RLU*/mg protein, which have been converted to ng/mg protein using a standard calibrated curve.

*Fluorescence assisted cell sorting*: The FACS study was carried out with a Calibur 345 flow cytometer equipped with a 488 nm laser and the BD CellQuest^TM^ Pro software. Details of the apparatus and the experimental procedure, using 48-well-plates, were reported recently [52]. The data were analyzed using the FlowJo LLC data software. Initially, the cells were gated using a forward scatter vs. side scatter (FSC vs. SSC) strategy to exclude any debris (low events), and then specifically analyzed by their 530 nm emission (FL1-H channel; the axis FL1-H shows the relative intensity of the GFP fluorescence). Transfection efficiencies (TE) were quantified by means of the percentage of cells in which GFP expression was observed (%GFP), and by the average intensity of fluorescence per cell (MFI, mean fluorescence intensity).

*Cell viability*: The cytotoxicity/cell viability was determined by a modified alamarBlue assay, following an experimental procedure reported previously [52]. Briefly, 1 mL of 10% (*v*/*v*) alamarBlue dye in Dulbecco’s modified Eagle medium, supplemented with 10% (*v*/*v*) FBS medium, was added to each well 48 h after transfection, and, after 2 h of incubation at 37 °C, assayed by measuring the absorbance at 570 and 600 nm, The cell viability (%) was obtained according to the expression: [(*A*_570_ − *A*_600_)_treated cells_/(*A*_570_ − *A*_600_)_untreated cells_] × 100, which correlates the absorbance data of treated and untreated cells [52]. Each sample was measured in three independent wells, and Lipo2000* was used as the positive control (1.5 μL of Lipo2000* per μg of DNA).

## 3. Results

### 3.1. Synthesis of the Gemini Lipid (C_3_(C_16_His)_2_)

Gemini-based lipids are perfect candidates for new synthetic vectors for DNA and RNA transfection [54]. Amino acid-based surfactants offer some advantages compared to the classical bisQuats gemini surfactants, in which the polar head consists of two quaternary ammonium groups: They can be synthesized from renewable materials using economical procedures and, generally, they exhibit low cytotoxicity [55,56]. Moreover, it has been reported that, if the polar head and the hydrophobic moiety are connected by an amide bond, the structure of the surfactants becomes more stable under both chemical and thermal conditions and displays high biodegradability [57]. Taking into account these advantages, a novel gemini cationic lipid based on the amino acid histidine has been synthesized. This compound, referred to as C_3_(C_16_His)_2_, consists of two *N*(*τ*),*N*(*π*)-bis(methyl)-histidine hexyl amides linked through a bridge of three –CH_2_– groups (Scheme 1). The palmitoyl fatty chain (C_16_) has been selected based on the structure–activity results relationship reported in the literature, which suggests that surfactants with C_16_–C_18_ alkyl chains exhibit superior transfection properties [58,59].

The general chemical structure of the target gemini lipid (C_3_(C_16_His)_2_), as well as the strategy adopted for its preparation, is outlined in Scheme 1. The synthesis of this new GCL was carried out in four steps. The first step involved the methylation of the two nitrogens of the imidazol ring of the *N*(α)-Cbz-histidine, using di-methyl sulfate (CH_3_)_2_SO_4_ dissolved in methanol as a methylating agent. The yield of the reaction was very high and the reaction mixture contained only the target intermediate and inorganic salts, which could be easily removed by filtration of the mixture in dry methanol. This bismethylated *N*(α)-Cbz-histidine was then reacted with hexadecyl amine to form a *N*(*α*)-Cbz-*N*(*τ*),*N*(*π*)-bis(methyl)-histidine hexadecyl amide intermediate. The synthesis involved treatment of the Z-protected histidine with BOP and DABCO. Our group has recently prepared single chain *N*(*α*)-Cbz-*N*(*τ*),*N*(*π*)-bis(methyl)-histidine alkyl amide surfactants using a different procedure [60]. The method used in the past provided very good yields; however, the purification of the C_16_ derivative was very problematic. The use of the coupling agent BOP to rapidly and efficiently activate the carboxylic group of the protected *N*(α)-Cbz-histidine led to the alkylated intermediate with 90% conversion in 3 h. Purification of this compound was then performed by washing the mixture with diethyl ether to remove the coupling agent. The Cbz group of the *N*-terminus was then removed via catalytic hydrogenation. Afterwards, the two carboxylic groups of tartaric acid were reacted with the free amine of the *N*(*τ*),*N*(*π*)-bis(methyl)-histidine hexadecyl amide using the same coupling agent, to form a gemini lipid. Preparative HPLC chromatography was employed to purify the obtained GCL. The chemical structure and purity of all the compounds, i.e., the intermediates and target GCL, was established by NMR spectroscopy (^1^H, ^13^C, and ^13^C-DEPT), FT-IR, and mass spectrometry (UPLC-MS). The spectral characterization of the C_3_(C_16_His)_2_ gemini lipid, reported in Figure S1 in the Supplementary Materials, confirmed the designed structure. In the ^13^C NMR spectrum, the signal for the terminal CH_3_ was observed at 14.5 ppm, and those of the two CH_3_ groups attached to the imidazolium nitrogen atoms at 34.1 and 36.4 ppm. The carbon of the asymmetric CH moiety was observed at 52.7, and those of the imidazolium ring at 123 and 138.4 ppm. The ^13^C NMR spectrum shows two signals corresponding to CONH groups, in contrast to the single chain precursor bearing only one amide group. The ^1^HNMR spectrum is also consistent with such a dimeric structure. The terminal CH_3_ appears as a triplet at 0.9 ppm, while the signals of the two CH_3_ groups attached to each imidazolium appear as two singlets at 3.8 and 3.9 ppm. The signal for the CH_2_ moiety of the spacer chain appears as a multiplet at 2.3 ppm, confirming the dimerization of the single chain intermediate. The FT-IR spectrum also agrees with the target structure, with the absorption frequencies for the C=O stretching band (amide I) around 1644 cm^−1^. Moreover, a broad amide N–H band at 3200 cm^−1^ indicated the existence of hydrogen bonded amide groups. The FT-IR spectrum also contains the frequencies corresponding to aromatic groups and long alkyl chains (Figure S1). The structure of this gemini was also established by UPLC-MS mass spectrometry, which showed a [M-Cl]^+2^ peak corresponding to the dipositive molecular ion lacking both chloride ions.

### 3.2. Biophysics of the C_3_(C_16_His)_2_/DOPE-pDNA Lipoplexes

**Figure 1:**
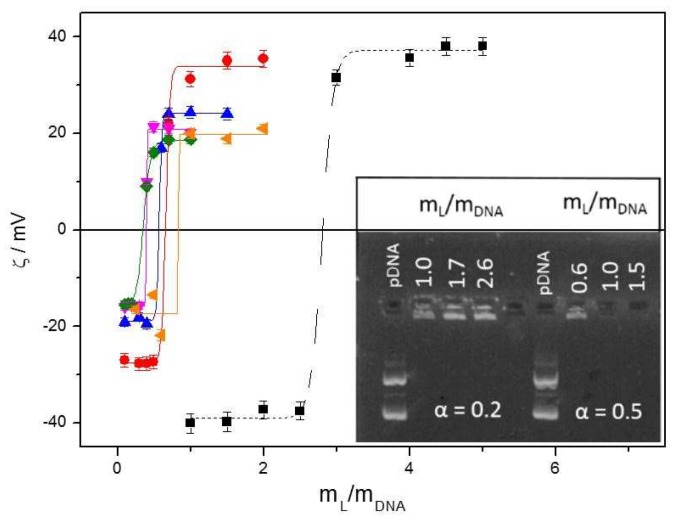
Plots of the zeta potential (*ζ*) against the *m_L_*/*m_DNA_* mass ratio in the C_3_(C_16_His)_2_/DOPE-pDNA lipoplexes at various mole fractions of the C_3_(C_16_His)_2_ cationic lipid in the C_3_(C_16_His)_2_/DOPE mixed lipid (*α* = 0.2, 0.4, 0.5, 0.6 and 0.7; red, blue, pink, orange and green, respectively) and of the C_3_(C_16_His)_2_/DOPE-ctDNA lipoplex at *α* = 0.5 (black). Inset: Electrophoresis agarose gel of the C_3_(C_16_His)_2_/DOPE-pDNA lipoplexes at various *m_L_*/*m_DNA_* mass ratios and *α* = 0.2 and 0.5.

Prior to biological studies to evaluate whether the presence of amino acid residues as cationic heads in the gemini cationic lipid (C_3_(C_16_His)_2_) was able to promote the transfection efficiency and bioavailability of plasmid DNA, a detailed physicochemical characterization of the C_3_(C_16_His)_2_/DOPE-pDNA lipoplexes was carried out. In this context, zeta potential and agarose gel electrophoresis (compaction assay) are fundamental tools to assess the electroneutrality of such a lipoplex. Figure 1 (inset) shows the electrophoresis agarose gel image for C_3_(C_16_His)_2_/DOPE-pDNA lipoplexes at two C_3_(C_16_His)_2_ molar compositions, (*α*) = 0.2 and 0.5, at various *m_L_*/*m_DNA_* = (*m*_*L*^+^_ + *m*_*L*^0^_)/*m_DNA_* mass ratios. In the first lane, used as the control, coiled and supercoiled forms of plasmid DNA are observed as two fluorescent bands. The absence of these fluorescent bands in the other lanes indicates that a full compaction of pDNA remained in the well. The minimum *m_L_*/*m_DNA_* mass relation at which all pDNA was effectively compacted was 1 and 0.6, at molar fractions of C_3_(C_16_His)_2_ in the C_3_(C_16_His)_2_/DOPE mixed lipid (*α*) of 0.2 and 0.5, respectively. In order to determine with better accuracy the mass relation value at which charge inversion of the C_3_(C_16_His)_2_/DOPE-pDNA lipoplex occurs, measurements of the zeta potential (*ζ*) were carried out at several mass ratios. Figure 1 reports the zeta potential data against *m_L_*/*m_DNA_* at different molar fractions (*α* = 0.2, 0.4, 0.5, 0.6, and 0.7); the fitted curves present the typical sigmoidal profile, where the lipoplexes display charge inversion at specific (*m_L_*/*m_DNA_*)*_ϕ_* values. The effective charges of the C_3_(C_16_His)_2_ cationic lipid (*q*_*eff*,*C*_3_(*C*_16_*His*)_2__ = 1.8 ± 0.1) and plasmid DNA (*q*_*eff*,*pDNA*_ = −0.3 ± 0.1) were determined from the electroneutrality data at varying molar fractions (*α*), using a procedure reported by us [27,51,61] and fully described in the Supplementary Materials. These values indicate that, while the C_3_(C_16_His)_2_ lipid presented almost the whole positive nominal charge (+2), pDNA, at physiological pH it remained in a supercoiled conformation [62,63] and exhibited only around 14% of its negative nominal charge (−2/*bp*). This fact is very advantageous, since a smaller amount of cationic vector is necessary to obtain a positive complex, thus reducing its potential cytotoxicity. The effective charge ratio of the C_3_(C_16_His)_2_/DOPE-pDNA lipoplex (*ρ_eff_*) between the positive C_3_(C_16_His)_2_/DOPE mixed lipid (*n*^+^) and the negative pDNA (*n*^−^) charges required for lipoplexes to be appropriate for transfection can be easily determined by using the C_3_(C_16_His)_2_ and pDNA effective charges previously obtained through the relation:

(1) $$\rho_{eff}=\frac{n^{+}}{n^{-}}=\frac{q_{eff,L}^{+}\left(m_{L^{+}}/M_{L^{+}}\right)}{q_{eff,DNA}^{-}\left(m_{DNA}/M_{DNA}\right)}$$

where *M*_*L*^+^_ and *M*_*DNA*_ are the molecular weight of the GCL and pDNA, respectively.

In addition, the size and structure of the lipoplexes, key parameters related to their effective biological activity, were determined by DLS and SAXS analyses. The hydrodynamic size (*D_h_*) and polydispersity index (PDI) values for C_3_(C_16_His)_2_/DOPE-pDNA complexes with two different pDNA plasmids (pEGFP-C3 and pCMV-Luc) are collected in Table 1 at two different molar fractions of the mixed lipid, and at two effective charge ratios of the lipoplex. The obtained sizes (*D_h_* = 135–185 nm), together with the low polydispersity indexes, indicate that the prepared lipoplexes are suitable to potentially cross the cell membrane.

**Figure 2:**
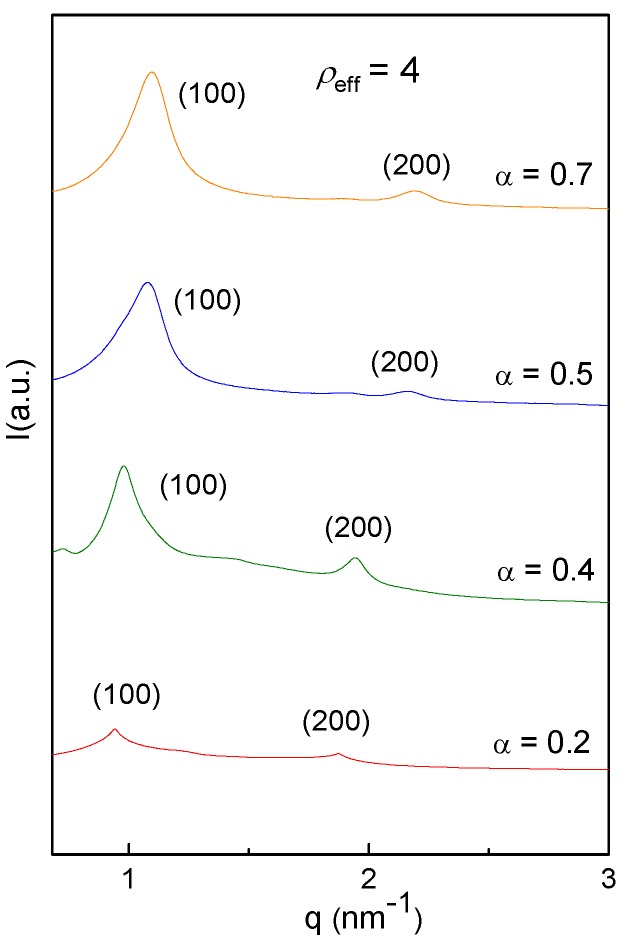
SAXS diffractograms of C_3_(C_16_His)_2_/DOPE-pDNA lipoplexes at an effective charge ratio *ρ_eff_* = 4 and several molar fractions of the C_3_(C_16_His)_2_ cationic lipid in the C_3_(C_16_His)_2_/DOPE mixed lipid (*α*).

**Scheme 2:**
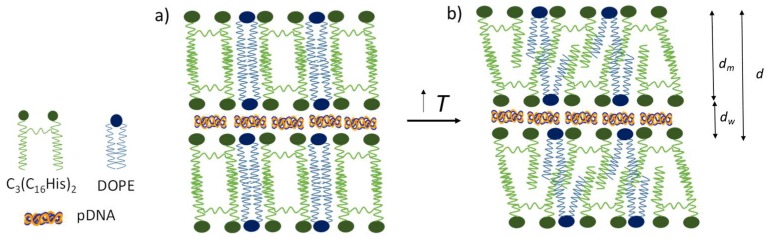
Lamellar structure of the C_3_(C_16_His)_2_/DOPE-pDNA lipoplex showing (**a**) the gel phase, and (**b**) the fluid phase of the C_3_(C_16_His)_2_/DOPE mixed lipid bilayer after reaching the gel-to-fluid transition temperature (*T_m_*). In the figure, the interlayer periodic distance (*d*) results from the sum of the lipid bilayer (*d_m_*) and aqueous monolayer (*d_w_*) thicknesses.

SAXS diffractograms were also recorded for C_3_(C_16_His)_2_/DOPE-pDNA lipoplexes, with the pEGFP-C3 plasmid at a wide range of molar compositions (*α* = 0.2, 0.4, 0.5, and 0.7) and effective charge ratios of the lipoplex (*ρ_eff_* = 1.5, 2.5, and 4). Figure 2, where the intensity at *ρ_eff_* = 4 is plotted vs. the momentum transfer vector (*q*), shows that the Bragg peaks in all the diffractograms correlate well with the Miller indexes (100) and (200), which are typical of a multilamellar (*L_α_*) lyotropic crystal phase (the plots for *ρ_eff_* = 1.5 and 2.5 are provided in Figure S2 in the Supplementary Materials). This lamellar structure can be explained as a sandwich-type phase, with alternating bilayers of C_3_(C_16_His)_2_/DOPE mixed lipid and the aqueous monolayer where the plasmid DNA and counterions are located (Scheme 2). The *q* factors of the Bragg peaks allow calculation of the interlayer periodic distance (*d* = 2*πn*/*q*_*n*00_), which results from the sum of the lipid bilayer (*d_m_*) and aqueous monolayer (*d_w_*) thicknesses. The obtained values for the periodicity (*d*) ranged from 5.7 to 6.7 nm, in agreement with those previously reported [52,64] for other mixed lipid systems formed by DOPE (18C) and a cationic lipid containing an alkyl chain of similar length (16C). Interestingly, a decrease of the interlayer distance *d* was observed (from 6.7 to 5.7 nm) with decreasing GCL molar fraction *α* (i.e., when the C_3_(C_16_His)_2_ content decreases and that of DOPE increases). This fact can be explained as a combination of: (a) a thinner bilayer (i.e., a smaller *d_m_*) as *α* increases, because C_3_(C_16_His)_2_ is slightly shorter than DOPE; and/or (b) a higher compaction of pDNA, leading to a reduction of *d_w_* with the increasing *α*. Assuming Tanford’s model [65,66,67] and keeping in mind the partial hydrophilic character of the C_3_(C_16_His)_2_ spacer, which contains -NH and C=O groups as well as 16 carbon atoms in each of the two hydrophobic chains, the mixed lipid bilayer (C_3_(C_16_His)_2_/DOPE) should have a thickness *d_m_* of around 3.8 nm, thus leaving 2–3 nm for the aqueous monolayer (*d_w_*), which is a space suitable to house the pDNA.

**Figure 3:**
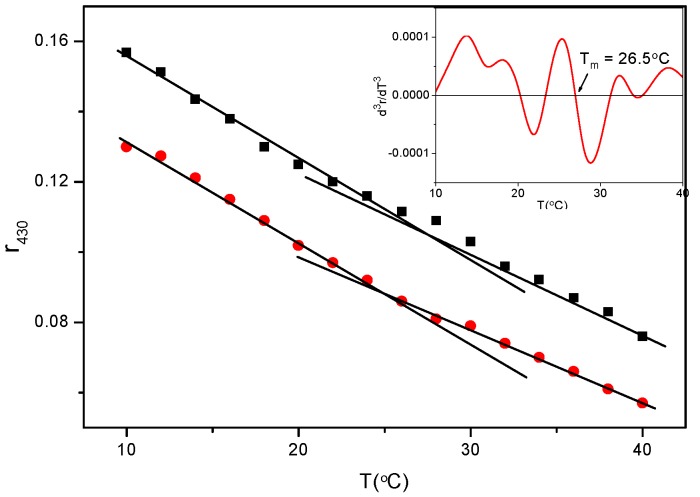
Fluorescence anisotropy at 430 nm (*r*_430_) of the DPH fluorescent probe against the temperature for the C_3_(C_16_His)_2_/DOPE-pDNA lipoplexes at an effective charge ratio *ρ_eff_* = 4 and two C_3_(C_16_His)_2_ cationic lipid molar fractions (*ρ* = 0.2 in red and *α* = 0.5 in black) in the C_3_(C_16_His)_2_/DOPE mixed lipid. The inset shows determination of the gel-to-fluid transition temperature (T*_m_*) following the Phillips method. Errors are less than 3%.

Fluorescence anisotropy may be correlated with the fluidity of a lipid bilayer [53,68], a property closely related to the transfection activity, since the fluidity of a lipoplex is directly related to its capability to cross the cellular membrane. The fluorescence anisotropy (*r*) was determined by rotation of a fluorescent probe, DPH, allowed in the bilayer of the lipoplexes. The degree of rotation of the DPH probe should increase with the lipid bilayer fluidity and, thus, the probe anisotropy should also decrease. Figure 3 reports the anisotropy data for the DPH probe allowed in the lipid bilayer of C_3_(C_16_His)_2_/DOPE-pDNA lipoplexes containing the pEGFP-C3 plasmid at two different molar fractions of the mixed lipid bilayer (*α* = 0.2 and 0.5) and an effective charge ratio of the lipoplex of *ρ_eff_* = 4, as a function of the temperature. The obtained *r* values indicate that the fluorescence anisotropy decreased with the temperature, which points to an increase in the lipid bilayer fluidity (Scheme 2). The gel-to-fluid transition temperature, determined as shown in the inset of Figure 3, following the Phillips method [69], was *T_m_* = (26 ± 1) °C at both *α* = 0.2 and 0.5, suggesting a fluid bilayer at physiological temperature (37 °C). It must be pointed that, in the whole temperature range analyzed, the anisotropy values were lower than 0.2, which is the typical threshold for a fluid membrane. Therefore, these anisotropy values were particularly low at physiological temperature, *r*_430_ = 0.05–0.08, rendering the C_3_(C_16_His)_2_/DOPE nanovector potentially attractive for application as a gene nanocarrier in vitro and in vivo assays.

**Figure 4:**
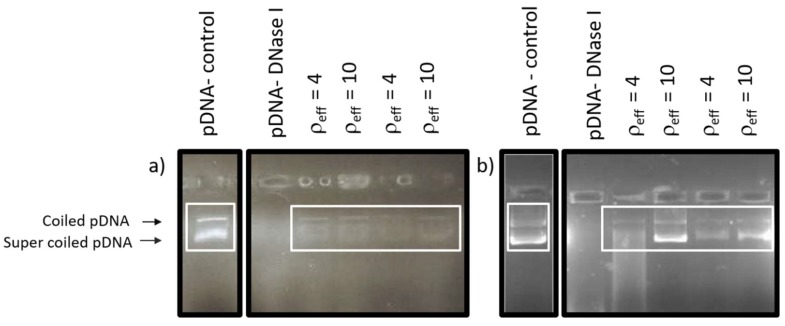
pDNA protection assay against degradation by DNase I (gel electrophoresis experiments): (**a**) pCMV-Luc plasmid, and (**b**) pEGFP-C3 plasmid. In both experiments: lane 1, pDNA control; lane 2, pDNA–DNase I; lanes 3–6, C_3_(C_16_His)_2_/DOPE-pDNA lipoplexes at two molar fractions of the C_3_(C_16_His)_2_ cationic lipid in the mixed lipid (lanes 3–4, *α* = 0.2; lanes 5–6, *α* = 0.5) and two effective charge ratios of the lipoplex lanes 3 and 5, *ρ_eff_* = 4; lanes 4 and 6, *ρ_eff_*= 10).

Bearing in mind that nucleic acids are susceptible to degradation by the DNases present in serum, the ability of this gene vector to efficiently compact and protect pDNA against degradation by DNase I was evaluated. Figure 4 reports the gel electrophoresis results for C_3_(C_16_His)_2_/DOPE-pDNA lipoplexes with two plasmids (pEGFP-C3 and pCMV-Luc), at two molar compositions (*α* = 0.2 and 0.5) and two effective charge ratios (*ρ_eff_* = 4 and 10). The naked plasmid was used as the control in the first lane (notice the characteristic fluorescent bands of DNA), whereas the second lane shows the DNA digested (and degraded) after DNase I addition (no fluorescent bands are present). Accordingly, the presence of DNA bands in the other lanes (3–6) confirms that C_3_(C_16_His)_2_/DOPE-pDNA successfully limited the access of DNase I to the compacted DNA. Notice that this protection was efficient for both plasmids, mostly at *ρ_eff_* = 10.

### 3.3. In Vitro Biological Activity of C_3_(C_16_His)_2_/DOPE-pDNA Lipoplexes

**Figure 5:**
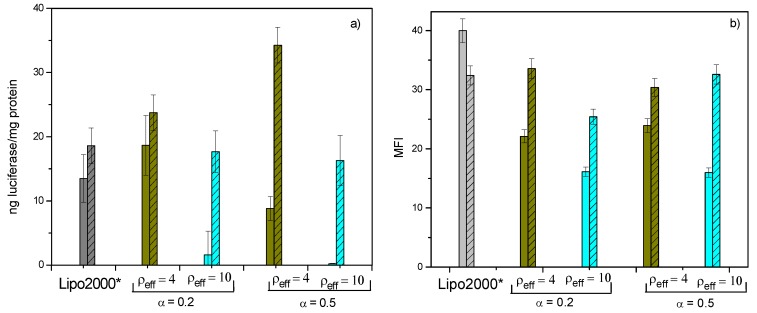
Transfection efficiency levels of C_3_(C_16_His)_2_/DOPE-pDNA lipoplexes in HeLa (solid bars) and COS-7 cells (dashed bars) at two molar fractions of the C_3_(C_16_His)_2_ cationic lipid in the C_3_(C_16_His)_2_/DOPE mixed lipid (*α* = 0.2 and 0.5): (**a**) in terms of ng of luciferase/mg of protein for plasmid pCMV-Luc, and (**b**) in terms of mean fluorescence intensity (MFI) for plasmid pEGFP-C3. The experiments were performed in the presence of 10% of fetal bovine serum (FBS). The green and blue bars correspond to effective charge ratios *ρ_eff_* = 4 and 10 of the lipoplex, respectively. Gray bar: Lipo2000* as the positive control. The data represent the mean ± SD of three wells and are representative of three independent experiments.

The C_3_(C_16_His)_2_/DOPE-pDNA lipoplexes were then evaluated in in vitro experiments, in order to assess the factors influencing their efficiency and safety and establish the optimum conditions for in vivo experiments. The transfection efficiency was evaluated on the COS-7 and HeLa cell lines in the presence of 10% of serum. Figure 5a shows the luciferase expression (for plasmid pCMV-Luc) in terms of ng of luciferase/mg of protein, as obtained from luminometry. Figure 5b and Figure S3 report the transfection efficiency (for plasmid pEGFP-C3), obtained by FACS and expressed in terms of mean fluorescence intensity per cell (MFI), in both cell lines (see Figure S3 of Supplementary Materials for the FACS results expressed in terms of %GFP). The experiments were carried out at two molar fractions of the mixed lipid, *α* = 0.2 and 0.5, and at two effective charge ratios of the lipoplex, *ρ_eff_* = 4 and 10. Additionally, all the results were compared against the universal positive control Lipofectamine 2000 (Lipo2000*). The results in Figure 5a show that, when transfecting plasmid pCMV-Luc, transfection efficacy was clearly better than that obtained with the control Lipo2000* at *ρ_eff_* = 4 at both *α* = 0.2 and 0.5 in COS-7 cells (especially at *α* = 0.5), and at *α* = 0.2 in HeLa cells; on the other hand, the results obtained at *ρ_eff_* = 10 remain comparable to that of the control Lipo2000* in the COS-7 cells and considerable lower in the HeLa cells, at the two molar fractions. Otherwise, Figure 5b shows that, when assessing transfection efficiency using plasmid pEGFP-C3: (a) in the COS-7 cells, all MFI results were comparable to those of the control, and interestingly, the MFI outcome at *ρ_eff_* = 4 and *α* = 0.2 was slightly better than that obtained with the control Lipo2000*; and (b) in the HeLa cells, MFI outcomes were higher at *ρ_eff_* = 4 than at *ρ_eff_* = 10 for both molar fractions (*α* = 0.2 and 0.5), but comparatively lower to those obtained for COS-7 and for the control Lipo2000*. The picture that emerges from the results in Figure 5 is that transfection efficiency is clearly dependent on the plasmid, the cell line, the effective charge ratio of the lipoplex, and the molar fraction of the mixed lipid constituting the gene carrier. In any case, the optimum formulations, i.e., with performances superior to those of Lipo2000*, were: (a) for plasmid pCMV-Luc, *ρ_eff_* = 4 at both molar fractions (*α* = 0.2 and 0.5) in COS-7 cells, and *ρ_eff_* = 4 at *α* = 0.2 in HeLa cells; and (b) for plasmid pEGFP-C3, *ρ_eff_* = 4 at *α* = 0.2 in COS-7 cells. Additionally, the higher levels of transfection achieved at the optimum formulations by the new synthetized lipids compared to the control Lipo2000* is an interesting result in terms of improving the efficacy of non-viral vectors for gene delivery, given that Lipo2000* is one of the best controls used to compare transfection efficacy. In an attempt to correlate these biological activity results with the structural information previously commented, it is remarkable that the lipoplexes herein studied are organized in multilamellar (L_α_) lyotropic liquid phases at the two molar fractions checked (i.e., *α* = 0.2 and 0.5), that show a similar fluid bilayer given the very low values of fluorescence anisotropy obtained in both cases. All this evidence seems to reinforce that both molar fractions (0.2 and 0.5) drive to formulations that are potentially valid for transfection purposes.

**Figure 6:**
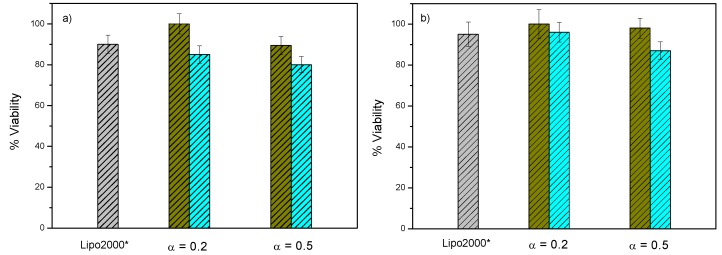
Cell viability of COS-7 cells in the presence of C_3_(C_16_His)_2_/DOPE-pDNA lipoplexes at two molar fractions of the C_3_(C_16_His)_2_ cationic lipid in the C_3_(C_16_His)_2_/DOPE mixed lipid (*α* = 0.2 and 0.5): (**a**) with pCMV-Luc plasmid and (**b**) with pEGFP-C3 plasmid. The green and blue bars correspond to effective charge ratios *ρ_eff_* = 4 and 10 of the lipoplex, respectively. Gray bar: Lipo2000* as the positive control. The data represent the mean ± SD of three wells and are representative of three independent experiments.

Furthermore, as vectors in biological studies are required to be both effective and safe for cells, the toxicity of the C_3_(C_16_His)_2_/DOPE-pDNA lipoplexes for the COS-7 and HeLa cell lines was tested by an alamarBlue assay. The results are reported in Figure 6 (and Figure S4 of Supplementary Materials) for both plasmids (pCMV-Luc and pEGFP-C3). All formulations exceeded a viability percentage of 80%, typically considered the threshold for cell safety, with values that in some cases reached 95% viability, even greater than those shown by the control Lipo2000*, thus confirming that the C_3_(C_16_His)_2_/DOPE gene carrier is a promising candidate for the transfection of both DNA plasmids, pEGFP-C3 and pCMV-Luc, in both cell lines, COS-7 and HeLa. It should be noted that the lack of toxicity indicates that the different transfection efficiency observed between both lipoplexes and the positive control Lipo2000* can be attributed to a different behavior in terms of interaction with the cell, which could be related to improved entry into the cell by the novel GCL lipid.

Several works [1,3,18,22,37,38,46,47] reported in the literature have involved lipoplexes that consist of a cationic lipid with one or two alkyl chains and monovalent or multivalent cationic imidazole or amino acid head groups, but gemini cationic lipids with histidine-based head groups have not previously been explored as potential non-viral vectors. Since the biological activities in the literature have been reported with plasmids and/or cell lines different than those used in the present work, the performances of the gene vector determined herein are compared with those of a lipoplex previously reported by us [33], which was used to transfect the pEGFP plasmid into HeLa cells at several molar fractions of the mixing lipids and charge effective ratios of the lipoplex, similar to those selected in the present work. The gemini cationic vector of that mixed system (bis(hexadecyl imidazolium) propane), referred as C_3_(C_16_Im)_2_), had a comparable gemini structure to that of the present work C_3_(C_16_His)_2_, and DOPE was present as a coadjuvant lipid in both systems. Thus, the C_3_(C_16_Im)_2_/DOPE-pDNA lipoplex showed MFI = 20 (at *α* = 0.2 and *ρ_eff_* = 4) and 75% cell viability in the HeLa cell line in presence of serum, while the results obtained in the present work for the C_3_(C_16_His)_2_/DOPE-pDNA lipoplex are MFI = 25 (at *α* = 0.2–0.5 and *ρ_eff_* = 4) and 95% cell viability. In conclusion, the substitution of two imidazole head groups by two histidine residues in the GCL structure provokes a slight improvement of transfection efficiency and a moderate increase of cell viability (in HeLa cells), confirming that amino acid residues in general, and histidine residues in particular, contribute to a better cellular uptake and endosomal release of DNA to cytoplasm, probably due to the protonation of the imidazole group of histidine at cellular pH [5].

**Figure 7:**
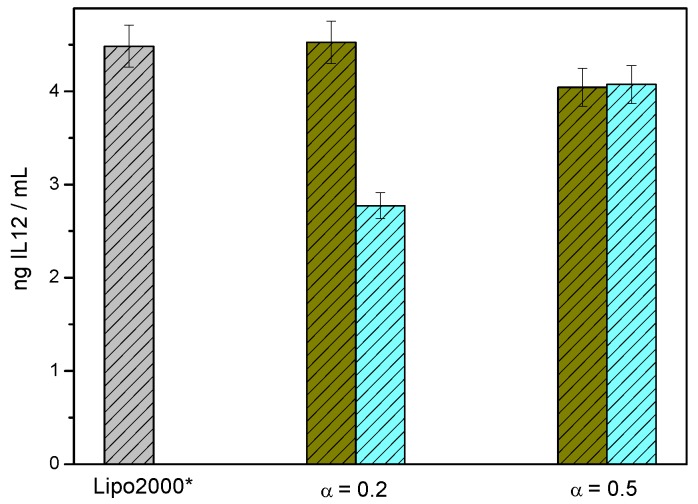
Transfection activity of C_3_(C_16_His)_2_/DOPE-pDNA lipoplexes in COS-7 cells at two molar fractions of the C_3_(C_16_His)_2_ cationic lipid in the C_3_(C_16_His)_2_/DOPE mixed lipid (*α* = 0.2 and 0.5) carrying plasmid pCMV-IL12. The green and blue bars correspond to effective charge ratios *ρ_eff_* = 4 and 10 of the lipoplex, respectively. Gray bar: Lipo2000* as the positive control. The data represent the mean ± SD of three wells and are representative of three independent experiments.

After considering these results, and in order to prove whether these new formulations could also enhance the transfection activity of complexes carrying a therapeutic gene, experiments with a very potent anti-tumoral cytokine gene (pCMV-interleukin-12) were performed. Figure 7 provides information on the activity of C_3_(C_16_His)_2_/DOPE-DNA (pCMV-IL12) in the COS-7 cell line, expressed in terms of ng pCMV-IL12 per mL. At *α* = 0.2 and *ρ_eff_* = 10 the transfection efficacy results are below those of the Lipo2000* control; however, those obtained at *α* = 0.5 and both *ρ_eff_* = 4 and 10, and at *α* = 0.2 and *ρ_eff_* = 4, are greater than or comparable to those of Lipo2000*. These results are not surprising in comparison with the data obtained using the reporter gene pCMV-Luc, given that different plasmids, even with a similar size, do not always display similar behavior in terms of transfection activity.

The overall in vitro results reported in the present work allow the conclusion that C_3_(C_16_His)_2_/DOPE-DNA lipoplexes may be interesting as nanocarriers for non-viral gene therapy applications, as illustrated through transfection efficiency with pEGFP-C3 and pCMV-Luc plasmids, and for nucleic acid immunotherapy with pCMV-IL12. Likewise, this system affords high cell viability independent of the compacted plasmid, being therefore a potential candidate for study in future in vivo experiments.

## 4. Conclusions

A novel biocompatible gemini cationic lipid (C_3_(C_16_His)_2_) bearing histidine residues was synthesized, and the mixed lipid together with the DOPE helper lipid was used as a gene vector for three DNA plasmids. The C_3_(C_16_His)_2_/DOPE-pDNA lipoplexes were characterized by biophysical and biological studies, and the delivery efficiency was evaluated by in vitro experiments. The zeta potential results revealed that, while the C_3_(C_16_His)_2_ lipid presented an effective charge (*q*_*eff*,*C*_3_(*C*_16_*His*)_2__ = 1.8 ± 0.1) close to the whole positive nominal charge (+2), the pDNA plasmid only exhibited around 14% of its negative nominal charge (−2/bp). Agarose gel electrophoresis assays confirmed that the C_3_(C_16_His)_2_/DOPE vector satisfactorily compacted and protected pDNA against DNase I degradation. DLS measurements showed that the formed C_3_(C_16_His)_2_/DOPE-pDNA complexes presented sizes ranging 120–190 nm, being thus very suited for efficient gene delivery. The SAXS diffractograms of the C_3_(C_16_His)_2_/DOPE-pDNA lipoplexes were consistent with a lamellar structure based in a sandwich-type phase, with alternating layers of C_3_(C_16_His)_2_/DOPE mixed lipids and an aqueous monolayer containing the pDNA and counterions. The low fluorescence anisotropy values indicated a fluid bilayer at physiological temperature, making the C_3_(C_16_His)_2_/DOPE nanovector potentially attractive for application as a gene carrier in in vitro and in vivo studies. The cytotoxicity studies showed that the C_3_(C_16_His)_2_/DOPE-pDNA complexes were well tolerated by both COS-7 and HeLa cells. The in vitro biological experiments allowed us to conclude that the optimum formulations of the C_3_(C_16_His)_2_/DOPE-pDNA lipoplexes are highly efficient and biocompatible as nucleic acid nanocarriers in COS-7 and HeLa cells, with results comparable or superior to those of the universal positive control Lipo2000*. In summary, results reported in this work prove that the C_3_(C_16_His)_2_/DOPE-pDNA lipoplex constitutes an interesting platform for in vitro gene delivery, with high cell bioavailability, and accordingly is a potential candidate for future in vivo applications.

## Figures and Tables

**Table 1:** Size *(D_h_*) and polydispersity index (PDI) values of the C_3_(C_16_His)_2_/DOPE-pDNA lipoplex at two effective charge ratios (*ρ_eff_*), and at two molar fractions of the C_3_(C_16_His)_2_ cationic lipid in the C_3_(C_16_His)_2_/DOPE mixed lipid, *α* = 0.2 and *α* = 0.5.

Lipoplex	*ρ_eff_* = 4		*ρ_eff_* = 10	
	*D_h_* (nm)	PDI	*D_h_* (nm)	PDI
*α* = 0.2				
C_3_(C_16_His)_2_/DOPE-pEGFP-C3	170	0.17	136	0.11
C_3_(C_16_His)_2_/DOPE-pCMV-Luc	177	0.27	119	0.15
*α* = 0.5				
C_3_(C_16_His)_2_/DOPE-pEGFP-C3	147	0.12	177	0.40
C_3_(C_16_His)_2_/DOPE-pCMV-Luc	154	0.19	185	0.26

Errors are less than 10%.

## Supplementary Materials

The following information is available online at http://www.mdpi.com/2079-4991/8/12/1061/s1. Details of the synthesis of intermediates and the gemini cationic lipid (C_3_(C_16_His)_2_); Figure S1: Characterization of the C_3_(C_16_His)_2_ gemini cationic lipid: ^1^H, ^13^C, and ^13^C-DEPT NMR spectra, and UPLC-MS profile; determination of the effective charges of the gemini cationic lipid and plasmid DNA; and additional results: zeta potential, electrophoresis gel assays for DNA protection; Figure S2: SAXS diffractograms of C_3_(C_16_His)_2_/DOPE-pDNA lipoplexes at effective charge ratios *ρ_eff_* = 1.5 and 2.5 and several molar compositions of the C_3_(C_16_His)_2_ cationic lipid in the C_3_(C_16_His)_2_/DOPE mixed lipid (*α*); Figure S3: Cell Viability of HeLa cells in the presence of C_3_(C_16_His)_2_/DOPE-pDNA lipoplexes, at two molar compositions of the cationic lipid in the mixed lipid (*α* = 0.2 and 0.5) with pCMV-Luc plasmid. Green and blue bars correspond to effective charge ratios *ρ_eff_* = 4 and 10 of the lipoplex, respectively. Gray bar: Lipo2000*, as positive control. The data represent the mean ± s.d. of three wells and are representative of three independent experiments; and Figure S4: Cell Viability of HeLa cells in the presence of C_3_(C_16_His)_2_/DOPE-pDNA lipoplexes, at two molar compositions of the cationic lipid in the mixed lipid (*α* = 0.2 and 0.5) with pCMV-Luc plasmid. Green and blue bars correspond to effective charge ratios *ρ_eff_* = 4 and 10 of the lipoplex, respectively. Gray bar: Lipo2000*, as positive control. The data represent the mean ± s.d. of three wells and are representative of three independent experiments.

## Abbreviations

C_3_(C_16_His)_2_	bis(*N*(τ),*N*(π)-bis(methyl)-histidine hexadecyl amide) propane
DOPE	1,2-dioleoyl-sn-glycero-3-phosphoethanolamine
DPH	Diphenylhexatriene fluorescent probe
FBS	Fetal Bovine Serum
Lipo2000*	Control Lipofectamine 2000 in presence of serum FBS
pCMV-Luc	Plasmid DNA encoding Luciferase
pCMV-IL12	Plasmid DNA encoding Interleukin-12
pEGFP-C3	Plasmid DNA encoding Green Fluorescent Protein
$q_{L^{+}}^{+}$	Positive effective charge of the cationic lipid
$q_{pDNA}^{-}$	Negative effective charge of plasmid DNA per base pair (bp)
