# Supplementary material for: A Gemini Cationic Lipid with Histidine Residues as a Novel Lipid-Based Gene Nanocarrier: A Biophysical and Biochemical Study
Source: Nanomaterials (Basel). 2018 Dec 16;8(12):1061. doi: 10.3390/nano8121061 (PMC6316511; doi:10.3390/nano8121061)
Supplement: Supplementary file 1 [file nanomaterials-08-01061-s001.pdf]

nanomaterials

Supplementary Materials for:

A Gemini Cationic Lipid with Histidine Residues as a Novel Lipid-Based Gene Nanocarrier: A Biophysical and Biochemical Study

María Martínez-Negro[1], Laura Blanco-Fernández[2], Paolo M. Tentori[1], Lourdes Pérez[3], Aurora Pinazo[3], Conchita Tros de Ilarduya[2], Emilio Aicart[1], and Elena Junquera[1,*]

[1] Grupo de Química Coloidal y Supramolecular, Departamento de Química Física, Facultad de Ciencias Químicas, Universidad Complutense de Madrid, 28040 Madrid, Spain; mmnegro@ucm.es (M.M.-N.); paolo.tentori@sns.it (P.T.); aicart@ucm.es (E.A.); junquera@ucm.es (E.J.)

[2] Departamento de Farmacia y Tecnología Farmacéutica, Facultad de Farmacia, Universidad de Navarra, Instituto de Investigación Sanitaria de Navarra (IdiSNA), 31008 Pamplona, Spain; lblancof@unav.es (L.B.-F.); ctros@unav.es (C.T.-I.)

[3] Dpto. Tecnología Química y Tensioactivos, IQAC-CSIC, 08034-Barcelona, Spain; lpmste@cid.csic.es (L.P.); apgste@cid.csic.es (A.P.)

Correspondence: junquera@ucm.es; Phone: +34913944131

1. Synthesis of the Cationic Gemini Lipid, bis(*N*(τ),*N*(π)-bis(Methyl)-Histidine Hexadecyl Amide) Propane ($C_3(C_{16}His)_2$)

The progress of the reactions and the purity of all compounds were followed by HPLC, model Merck-Hitachi D-2500, using a L-4250 UV-VIS detector at 215 nm. A Lichrospher 100 CN (propylcyano) 5 μm, 250 × 4 mm column was used [1]. A gradient elution profile was employed from an initial solvent ratio of A/B 75/25 (by volume) to a final one of 5/95 after 24 min, where solvent A was 0.1% (v/v) trifluoro acetic acid (TFA) in H_2O and solvent B was 0.085% of TFA in H_2O/CH_3CN 1:4. The flow-rate through the column was 1.0 mL/min. HPLC methodology was used to purify the target surfactants. HPLC was carried out by a Waters HPLC system equipped with a Kromasil 100 C8 5 μm 25 × 2.12 column. The crude product (500 mg dissolved in 5 mL of methanol) was loaded on the preparative column. The solvent system consisted of 0.1% TFA in water (solvent A) and 0.1% TFA in acetonitrile (solvent B). The compounds were eluted at a flow rate of 20 mL/min with a linear gradient from mixture A:B (60:40, v/v) to A:B (0:100, v/v) in 20 min. The absorbance of the eluate was measured at 215 nm. The structures of the target compounds were characterized by ^1H and ^{13}C NMR spectroscopy recorded on a Varian spectrometer at 499.803 (^1H) and 125.233 (^{13}C) MHz, respectively, using the deuterium signal of the solvent as the lock. Chemical shifts (δ) are reported in ppm downfield from tetramethylsilane (TMS).[2] The NMR experiments were performed in 5-mm tubes (0.6 mL sample) using a 5-mm indirect broadband probe. ^{13}C NMR spectra were recorded under composite decoupling to eliminate ^{13}C-^1H coupling. Distortionless enhancement by polarization transfer (DEPT) spectra was recorded to phase up and down the CH/CH_3 and CH_2 signals, respectively. Mass spectra with fast atom

bombardment (FAB) or electrospray techniques were recorded on a VG-QUATTRO from Fisons Instruments [2].

Synthesis of *N*(α)-Cbz-*N*(π),*N*(τ)-bis(methyl)-L-histidine (ZDMH): *N*(α)-Cbz-histidine (5 mmol) was dissolved in methanol (75 mL) and K_2CO_3 was added up to pH = 10–11. The solution was cooled with ice to 0 °C and $(CH_3)_2SO_4$ (15 mmol) was added dropwise to the stirred solution. The reaction mixture was stirred at room temperature for 4 h maintaining the same pH value. Then, HCl was added up to pH = 6. The solvent was removed and the crude material was extracted with dried ethanol to remove the salts. After freeze-drying, a white solid was obtained with 99% purity by HPLC.

Synthesis of *N*(α)-Cbz-*N*(π),*N*(τ)-bis(methyl)-L-histidine hexadecyl amide ($ZDMHNHC_{16}$): *N*(α)-Cbz-*N*(π),*N*(τ)-bis(methyl)-L-histidine (4 mmol) was dissolved in DMF and hexadecyl amine (4 mmol), BOP (4 mmol), and DABCO (6 mmol) were added. The reaction progress was monitored by analytical HPLC. After completion of the reaction (3 h), the DMF was removed by vacuum and the reaction mixture was washed several times with diethyl ether to remove the BOP reagent. The products were identified by HPLC, ^{1}H and ^{13}C NMR spectroscopy, and mass spectrometry.

$ZDMHNHC_{16}$. Yield: 85%. **^{1}H NMR**: δ_H (CD_3OD), 0.87–092 [t, 3H, –C$\underline{H_3}$, alkyl chain], 1.32–1.66 [m, 28H, –C$\underline{H_2}$–, alkyl chain], 2.93–3.32 [m, 4H, –C$\underline{H_2}$– histidine, CONH–$\underline{CH_2}$], 3.77, 3.88 [2s, 6H, 2 –C$\underline{H_3}$,], 4.42–4.45 [m, 1H, –C$\underline{H}$–, histidine], 5.04–5.12 [2H, –C$\underline{H_2}$–, Cbz group], 7.26–7.28 [m, 6H, -C$\underline{H}$-, Cbz group and imidazole group], 8.75 [s, 1H, –C$\underline{H}$–, imidazole group] ppm. **^{13}C NMR**: δ_C (CD_3OD), 14.4 [–$\underline{C}H_3$, alkyl chain], 23.7–33.0, [–$\underline{C}H_2$–, alkyl chain], 34.0, 36.3 [–$\underline{C}H_3$, imidazole group], 40.5 [–CONH–$\underline{C}H_2$–], 54.3 [–$\underline{C}$H–,

histidine], 67.7 [–CH_2–, Cbz group], 123.0 [–CH–, imidazole], 129.0–129.6 [–CH–, Cbz group, –C–, imidazole group], 133.4 [–C–, Cbz group], 138.2 [–CH–, imidazole group], 158.1 [–COO–, Cbz group], 171.8 [–CO–NH–] ppm.

Synthesis of *N*(π),*N*(τ)-bis(methyl)-L-histidine hexyl amide ($DMHNHC_{16}$): Pure *N*(α)-Cbz-*N*(π),*N*(τ)-bis(methyl)-L-histidine hexadecyl amide (0.0035 mol) was dissolved in 30 mL methanol/HCl (mol HCl/mol histidine derivative = 1.2) and Pd in activated charcoal (10% Pd) was added as the catalyst. The reaction was carried out at atmospheric pressure. At the end of the reaction (30 min), the catalyst was filtered off through celite. The solvent was evaporated under reduced pressure. The solid was dissolved in water and lyophilized. Finally, the product was subjected to ion exchange chromatography on a Macro-Prep High S support-exchange resin column to isolate the pure dichloride salt, as identified by HPLC, ^1H and ^{13}C NMR spectroscopy, and mass spectrometry.

$DMHNHC_{16}$: Yield: 90%. **HPLC,** rt = 16.46 min. **MW** (calculated for $C_{24}H_{48}N_4OCl_2$) 497.6 g/mol. **^1H NMR**: δ_H (CD_3OD), 0.90 [t, 3H, –CH_3, alkyl chain], 1.04–1.67 [m, 28H, –CH_2–, alkyl chain], 3.20–3.44 [m, 4H, –CH_2 histidine and –CONH–CH_2–], 3.89, 3.90 [2s, 6H, 2 –CH_3], 4.22 [t, 1H, –CH-, histidine], 7.54 [s, 1H, –CH–, imidazole group], 8.92 [s, 1H, –CH–, imidazole group] ppm. **^{13}C NMR**: δ_C (CD_3OD), 14.4 [–CH_3, alkyl chain], 23.7–33.0, [–CH_2–, alkyl chain], 34.3, 36.6 [–CH_3– imidazole group], 40.8 [CONH–CH_2–], 52.6 [–CH–, histidine], 124.5 [–CH–,imidazole group], 130.2 [–C–, imidazole group], 139.2 [–CH–, imidazole group], 168.2 [–CO–NH–] ppm. **UPLC-MS** (*m*/*z*) calculated for $C_{24}H_{48}N_4OCl_2$ $[M-2Cl]^+$ 407.3; found 407.

Synthesis of gemini cationic lipid bis(*N*(τ),*N*(π)-bis(methyl)-L-histidine hexadecyl amide) propane ($C_3(C_{16}His)_2$). A solution of **$DMHNHC_{16}$** (3 mmol) in 50 mL of CH_2Cl_2 was treated with 6 mmol of DABCO. Then, 1.5 mmol of glutaric acid was added under stirring until total solubilization of the compounds. Finally, 3 mmol of BOP was added and the mixture was stirred for 3 h. After complete conversion of the starting materials, the mixture was cooled to 0 °C and the crude precipitate was isolated by filtration. The solid was washed with diethyl ether. Purification of the amino acid-based product was achieved by preparative HPLC on a C8 reversed-phase column. All collected fractions were analyzed directly by HPLC on a 100 CN (Propylcyano) column to determine the purity (%) and the combined pure portions were dried under vacuum.

$C_3(C_{16}His)_2$. Yield: 75%. **HPLC**, rt = 23.01 min. **MW:** 983.33 g/mol. **ESI-MS:** *m*/*z* = 455 $(M\text{-}H)/2^+$. **^{1}H NMR**: δ_H (CD_3OD), 0.90 [t, 6H, –C$\underline{H_3}$–, alkyl chain], 1.05–1.64 [m, 56H, –C$\underline{H_2}$–, alkyl chain], 1.83 [m, 2H, –C$\underline{H_2}$–, spacer chain], 2.23 [m, 4H, –C$\underline{H_2}$–CONH–, spacer chain], 2.93-3.28 [m, 8H, –CONH–C$\underline{H_2}$–, alkyl chain, C$\underline{H_2}$ histidine], 3.89 [12H, 4 –C$\underline{H_3}$, methylimidazolium group], 4.68 [m, 2H, –C$\underline{H}$-, histidine], 7.34- 7.42 [2s, 2H, -C$\underline{H}$-, methylimidazolium group], 8.85 [2H, –C$\underline{H}$–, methylimidazolium group] . **^{13}C NMR**: δ_C (CD_3OD), 14.4 [$\underline{C}H_3$–, alkyl chain], 23.7–40.7 [–$\underline{C}H_2$–, alkyl chain, –$\underline{C}H_2$–, spacer chain, –CONH–$\underline{C}H_2$, alkyl chain], 34.3, 36.4 [–$\underline{C}H_3$–, methylimidazolium group], 52.8 [–$\underline{C}$H–, histidine], 123.1 [–$\underline{C}$H–, methylimidazolium group], 133.3 [–$\underline{C}$–, methylimidazolium group], 138.42 [–$\underline{C}$H–, methylimidazolium group], 172.3 [CH_2–$\underline{C}$O–NH–], 175.4 [CH–$\underline{C}$O–NH–] ppm. **FT-IR** (4000-450 cm^{-1}): 3064 cm^{-1} [ν(Ar-H)]; 2916 cm^{-1}, 2849 cm^{-1} [ν(C-H)]; 3275 cm^-

[1] [ν(N-H) amide]; 1644 cm^{-1} [ν(C=O) amide I]; 1537 cm^{-1} [ν(C=C)] ; 1158 cm^{-1} [ν(C-C)]; 719 cm^{-1} [ν($-CH_2-$) n>4].

2. Determination of the Effective Charges of the Gemini Cationic Lipid (GCL) and Plasmid DNA (pDNA)

The composition of a mixed lipid used as gene vector is given as the molar fraction (α) of the cationic lipid, while in the case of the lipoplex, the composition may be given in terms of: a) the total lipid to DNA mass ratio, expressed as $m_L / m_{DNA} = (m_{L^+} + m_{L^0}) / m_{DNA}$, where m_L, m_{L^+}, m_{L^0}, and m_{DNA} are the mass of the total mixed lipid, GCL, DOPE, and DNA, respectively, and b) the effective charge ratio (ρ_{eff}) expressed as the ratio between the charges of positive GCL and negative DNA phosphate groups. All these quantities are related by the following two equations:

$$\alpha = \frac{m_{L^+} / M_{L^+}}{(m_{L^+} / M_{L^+}) + (m_{L^0} / M_{L^0})} \quad (1)$$

$$\rho_{eff} = \frac{n^+}{n^-} = \frac{q^+_{eff,\,L}(m_{L^+} / M_{L^+})}{q^-_{eff,\,DNA}(m_{DNA} / M_{DNA})} \quad (2)$$

where n^+ and n^- are the number of moles of positive and negative charges of GCL and DNA; $q^+_{eff,\,L}$ and $q^-_{eff,\,DNA}$ are the effective charges of GCL and pDNA per bp; and M_{L^+} and M_{DNA} are the molecular weight of the GCL and pDNA per bp, respectively. There is a particular $(m_{L^+} + m_{L^0}) / m_{DNA}$ lipoplex composition where the positive and negative charges balance (ρ_{eff} = 1). This particular value, known as electroneutrality ratio $\left((m_{L^+} + m_{L^0}) / m_{DNA}\right)_\phi$ is characteristic of the lipoplex and marks the lower limit from which the lipoplex becomes a potentially cell transfecting agent, since efficient cell

transfection needs net positively charged lipoplexes capable of crossing the negatively charged cell membrane [3]. Zeta potential (ζ), related to the net charge of the lipoplex, is the best physicochemical property to determine this electroneutrality ratio. Figure 1 of the MS show plots of ζ vs. (m_L / m_{DNA}) at several α values for the GCL used in this work. The electroneutrality ratio of the lipoplex can be determined as the m_L / m_{DNA} where a sign inversion on the charge on the ζ sigmoidal plots is detected. This value is related to the quantities of Equations (1-2) by:

$$\left(\frac{m_L}{m_{DNA}}\right)_{\Phi} = \left(\frac{m_{L^+} + m_{L^0}}{m_{DNA}}\right)_{\phi} = \frac{q^-_{DNA}[\alpha M_{L^+} + (1-\alpha) M_{L^0}]}{q^+_L \alpha M_{DNA}} \quad (3)$$

Studies reported in literature have shown that commercial linear DNA, as calf thymus DNA, or so on, has its negative charge totally available for the cationic lipid, i.e., $q_{linear\,DNA} = -2$ per base pair. But experiments reported by our group [4-7] have demonstrated that, at physiological conditions, plasmid DNA remains in a supercoiled conformation [8,9] rendering a much less negative charge than its nominal one $(q^-_{eff,\,pDNA} << -2/bp)$. For that reason, the first step of any biophysical study must start with the determination of the effective charge of both, the cationic lipid $(q^+_{eff,\,L})$ and the pDNA $(q^-_{eff,\,DNA})$.

Initially, the effective charge of the GCL $(q^+_{eff,\,L})$ can be determined for a certain GCL composition (α) of the mixed lipid vector, from Equation (3) using the $(m_L / m_{DNA})_{\Phi}$ value of the GCL/DOPE-linear DNA lipoplex experimentally measured from zeta potential (black curve in Figure 1 of the MS), and assuming that $q_{linear\,DNA} = -2$/bp. Once the effective charge of the GCL $(q^+_{eff,\,L})$ has been obtained, the determination of the electroneutrality value $(m_L / m_{DNA})_{\Phi}$ for the GCL/DOPE-pDNA lipoplex containing the plasmid DNA in identical

mixed lipid composition (α) (coloured curves in the Figure 1 of the MS) permits to obtain the effective charge of pDNA $(q^{-}_{eff,DNA})$ at each molar fraction of the mixed lipid (α) from rearranged Equation (3) as follows:

$$q^{-}_{eff,DNA} = \left(\frac{m_L}{m_{DNA}}\right)_{\Phi} \left(\frac{q^{+}_{L^+}\alpha M_{DNA}}{\alpha M_{L^+} + (1-\alpha) M_{L^0}}\right) \quad (4)$$

Once the effective charges of the cationic lipid and plasmid DNA are correctly determined, the effective charge ratio (ρ_{eff}) of the lipoplex at any molar fraction (α) of the mixed lipid may be calculated by substituting $(q^{+}_{eff,L})$ and the pDNA $(q^{-}_{eff,DNA})$ in Equation (2). The effective charge ratio of the lipoplex (ρ_{eff}) is the key to prepare lipoplexes with appropriate formulations to be used in biological studies, i.e., with a net positive charge.

References

1. Bustelo, M.; Pinazo, A.; Manresa, M.A.; Mitjans, M.; Vinardell, P.; Pérez, L. Monocatenary histidine-based surfactants: Role of the alkyl chain length in antimicrobial activity and their selectivity over red blood cells. *Colloids Surf. A* **2017**, *532*, 501-509.
2. Pinazo, A.; Angelet, M.; Pons, R.; Lozano, M.; Infante, M.R.; Perez, L. Lysine-bisglycidol conjugates as novel lysine cationic surfactants. *Langmuir* **2009**, *25*, 7803-7814.
3. Dias, R.S.; Lindman, B. *DNA Interaction with Polymers and Surfactants*; Wiley & Sons: Hoboken, NJ, 2008.
4. Muñoz-Úbeda, M.; Misra, S.K.; Barrán-Berdón, A.L.; Aicart-Ramos, C.; Sierra, M.B.; Biswas, J.; Kondaiah, P.; Junquera, E.; Bhattacharya, S.; Aicart, E. Why is less cationic lipid required to prepare lipoplexes from plasmid DNA than linear DNA in gene therapy? *J. Am. Chem. Soc.* **2011**, *133*, 18014-18017.
5. Barrán-Berdón, A.L.; Misra, S.K.; Datta, S.; Muñoz-Úbeda, M.; Kondaiah, P.; Junquera, E.; Bhattacharya, S.; Aicart, E. Cationic gemini lipids containing polyoxyethylene spacers as improved transfecting agents of plasmid DNA in cancer cells. *J. Mater. Chem. B* **2014**, *2*, 4640-4652.
6. Martinez-Negro, M.; Guerrero-Martinez, A.; Garcia-Rio, L.; Domenech, O.; Aicart, E.; de Ilarduya, C.T.; Junquera, E. Multidisciplinary approach to the transfection of plasmid DNA by a nonviral nanocarrier based on a gemini-bolaamphiphilic hybrid lipid. *ACS Omega* **2018**, *3*, 208-217.
7. Misra, S.K.; Muñoz-Úbeda, M.; Datta, S.; Barrán-Berdón, A.L.; Aicart-Ramos, C.; Castro-Hartmann, P.; Kondaiah, P.; Junquera, E.; Bhattacharya, S.; Aicart, E. Effects of a

delocalizable cation on the headgroup of gemini lipids on the lipoplex-type nano-aggregates directly formed from plasmid DNA. *Biomacromolecules* **2013**, *14*, 3951-3963.
8. Lyubchenko, Y.L.; Shlyakhtenko, L.S. Visualization of supercoiled DNA with atomic force microscopy in situ. *Proc. Natl. Acad. Sci. U.S.A.* **1997**, *94*, 496-501.
9. Foldvari, M.; Badea, I.; Wettig, S.; Verrall, R.; Bagonluri, M. Structural characterization of novel gemini non-viral DNA delivery systems for cutaneous gene therapy. *J. Exp. Nanosci.* **2006**, *1*, 165-176.

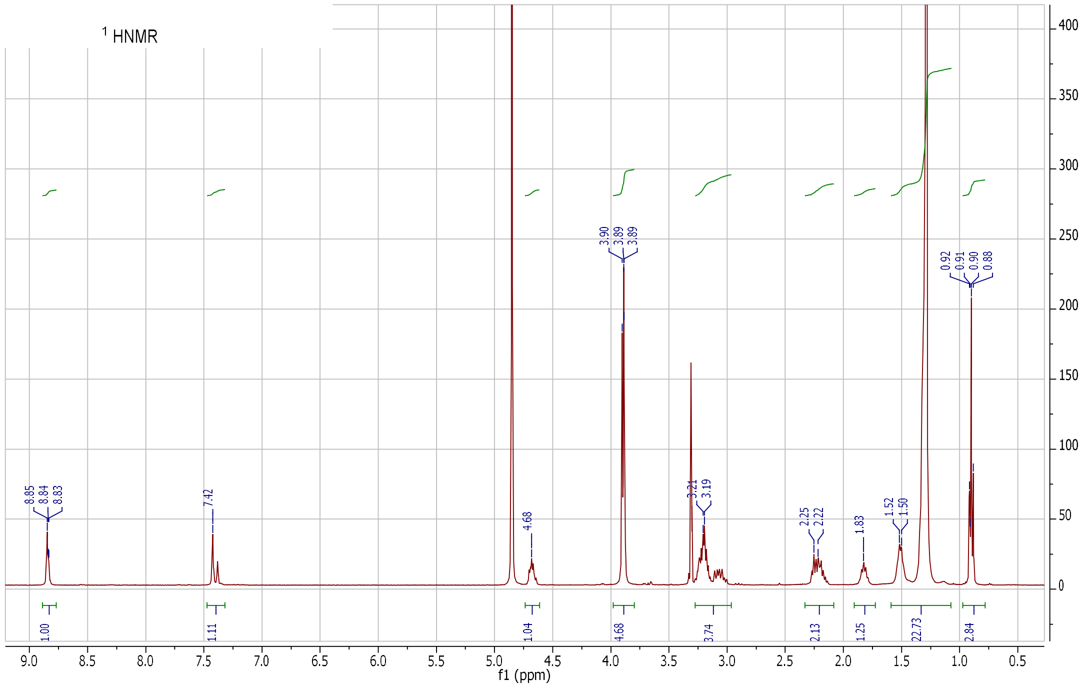
1 HNMR
8.85 8.84 8.83
7.42
4.68
3.90 3.89 3.89
3.21 3.19
2.25 2.22
1.83
1.52 1.50
0.92 0.91 0.90 0.88
1.00
1.11
1.04
4.68
3.74
2.13
1.25
22.73
2.84
f1 (ppm)

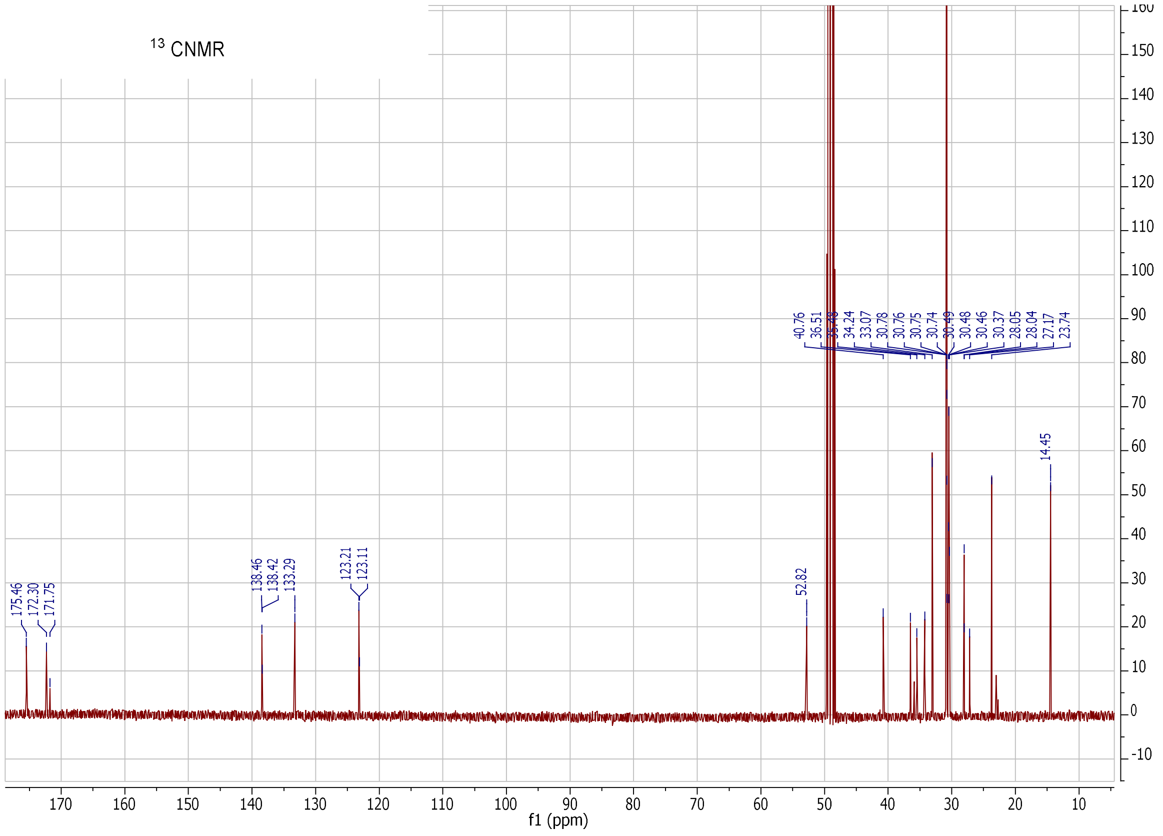
13 CNMR
175.46 172.30 171.75
138.46 138.42 133.29
123.21 123.11
52.82
40.76 36.51 34.24 33.07 30.78 30.76 30.75 30.74 30.48 30.46 30.37 28.05 28.04 27.17 23.74
14.45
f1 (ppm)

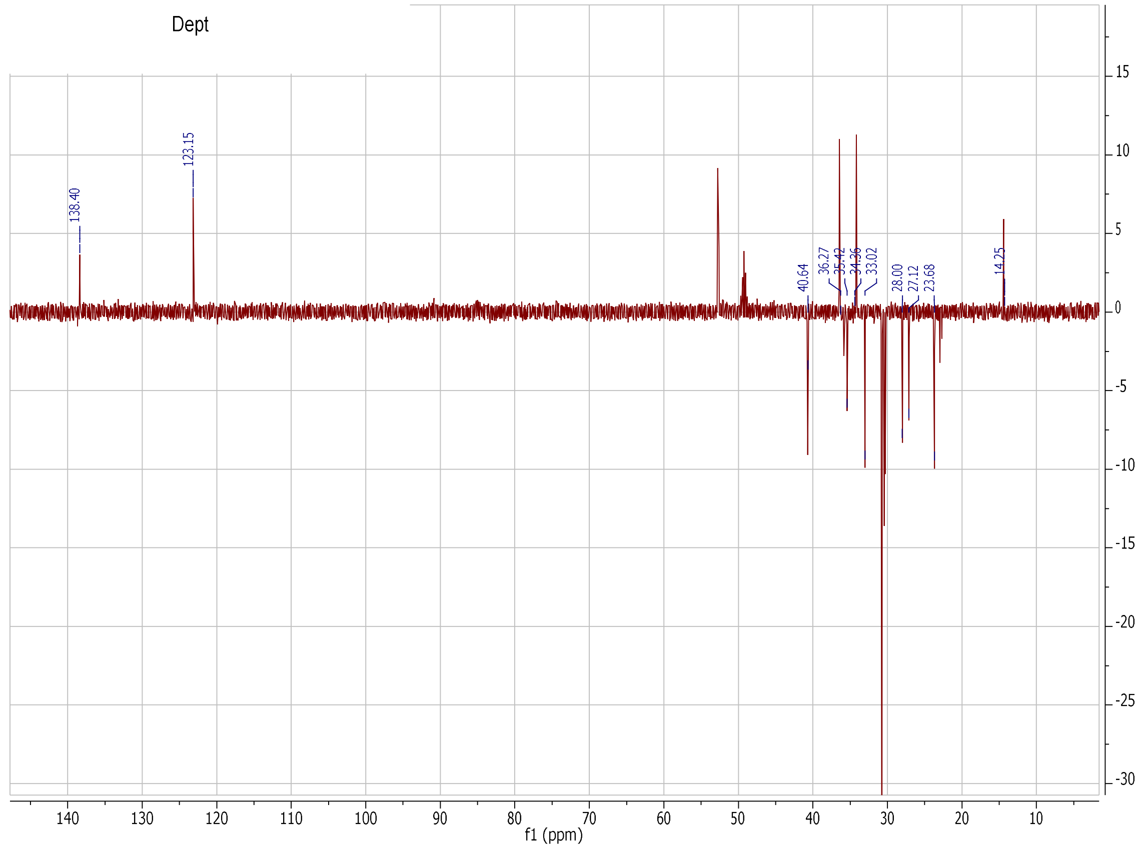
Dept
138.40
123.15
40.64
36.27
35.42
34.36
33.02
28.00
27.12
23.68
14.25
f1 (ppm)

FT_IR

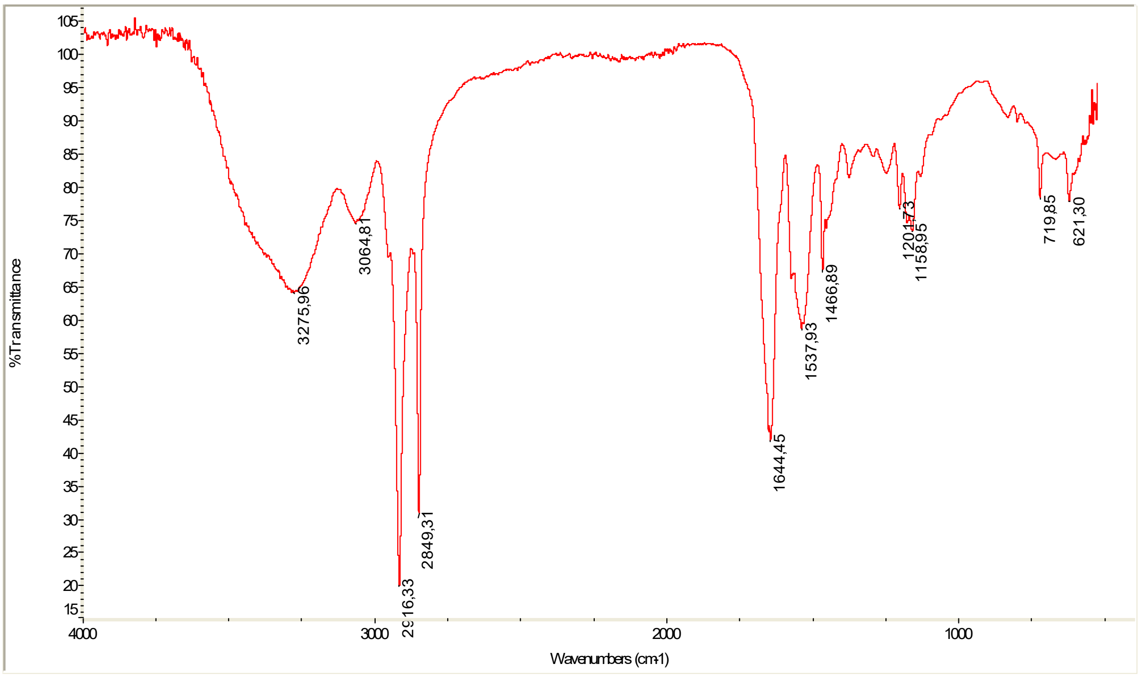
%Transmittance
Wavenumbers (cm-1)
3275,96
3064,81
2916,33
2849,31
1644,45
1537,93
1466,89
1201,73
1158,95
719,85
621,30

UPLC-MS

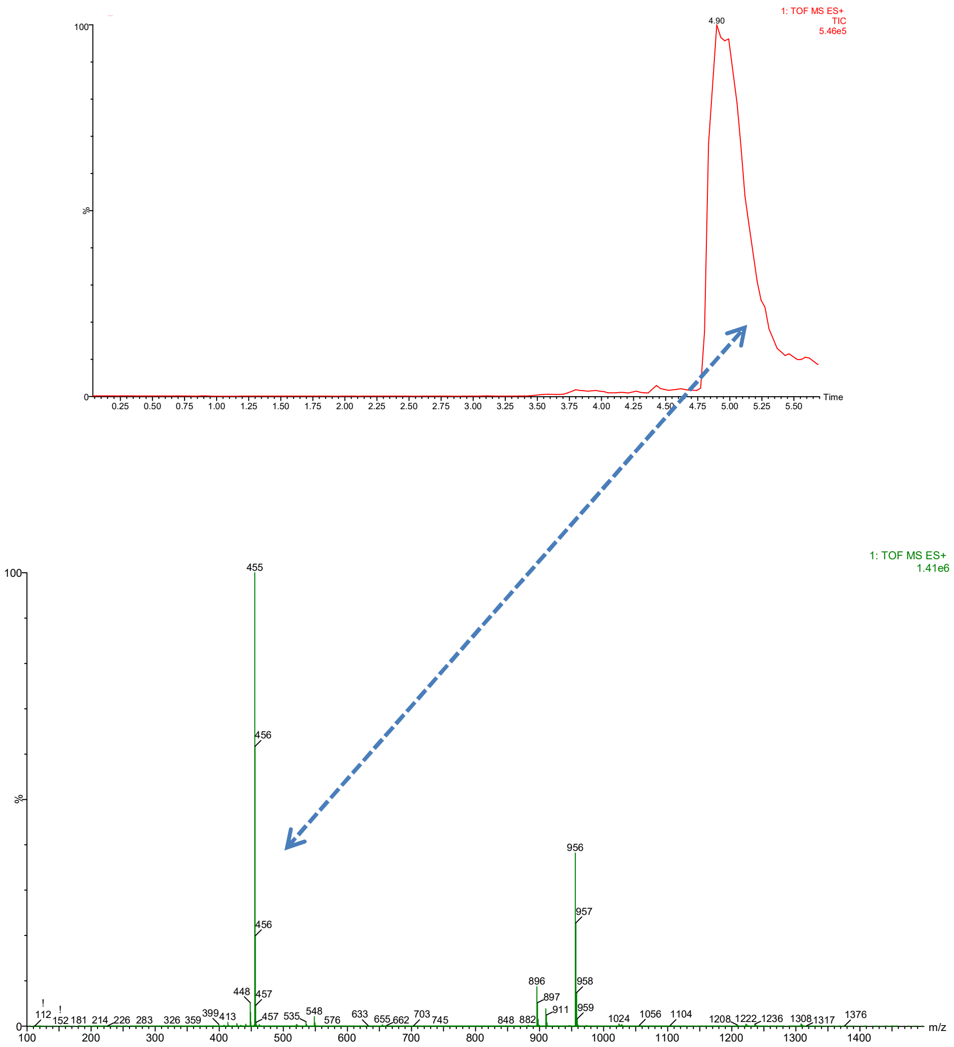

Figure S1. Characterization of the $C_3(C_{16}His)_2$ gemini cationic lipid: ^1H, ^{13}C, and ^{13}C-DEPT NMR spectra, FT-IR, and UPLC-MS profile.

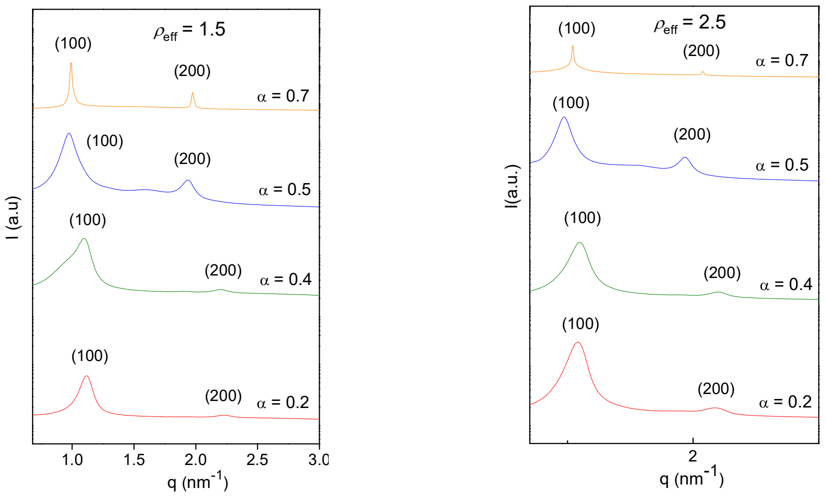

Figure S2. SAXS diffractograms of $C_3(C_{16}His)_2$/DOPE-pDNA lipoplexes at effective charge ratios ρ_{eff} = 1.5 and 2.5 and several molar compositions of the $C_3(C_{16}His)_2$ cationic lipid in the $C_3(C_{16}His)_2$/DOPE mixed lipid (α).

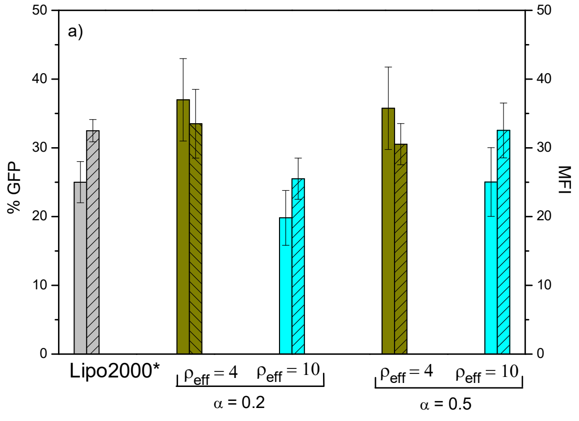

Figure S3. Transfection efficiency levels of $C_3(C_{16}His)_2$/DOPE-pDNA lipoplexes in COS-7 cells at two molar compositions of the $C_3(C_{16}His)_2$ cationic lipid in the $C_3(C_{16}His)_2$/DOPE mixed lipid (α = 0.2 and 0.5) in terms of % GFP (solid bars) and MFI (dashed bars) for plasmid pEGFP-C3. The experiments were performed in the presence of 10% of serum (FBS). The green and blue bars correspond to effective charge ratios ρ_{eff} = 4 and 10 of the lipoplex, respectively. Gray bar: Lipo2000* as the positive control.

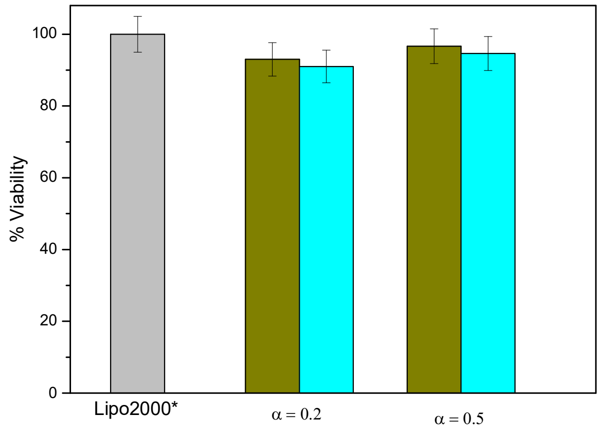

Figure S4. Cell Viability of HeLa cells in the presence of $C_3(C_{16}His)_2$/DOPE-pDNA lipoplexes, at two molar compositions of the cationic lipid in the mixed lipid (α = 0.2 and 0.5) with pCMV-Luc plasmid. Green and blue bars correspond to effective charge ratios ρ_{eff} = 4 and 10 of the lipoplex, respectively. Gray bar: Lipo2000*, as positive control. The data represent the mean ± s.d. of three wells and are representative of three independent experiments.
